# Communication in oncology between healthcare providers, patients, the scientific community, and the media: recommendations from the Italian Association of Medical Oncology (AIOM)

**DOI:** 10.1007/s00520-024-08786-8

**Published:** 2024-08-26

**Authors:** Rossana Berardi, Alessandro Parisi, Marco Maruzzo, Marco Bellani, Giordano Domenico Beretta, Mauro Boldrini, Luigi Cavanna, Stefania Gori, Elisabetta Iannelli, Anna Maria Mancuso, Massimiliano Marinelli, Vera Martinella, Michele Musso, Roberto Papa, Antonio Russo, Valentina Tarantino, Mirella Taranto, Saverio Cinieri

**Affiliations:** 1 Medical Oncology, Azienda Ospedaliera Universitaria (AOU) Delle Marche, Università Politecnica Delle Marche, Ancona, Italy; 2 Oncology Unit 1, Department of Oncology, Istituto Oncologico Veneto IOV-IRCCS, Padova, Italy; 3 Psycho-Oncology Unit, Department of Medicine and Innovation Technology, University of Insubria, Varese, Italy; 4 UOC Oncologia Medica, ASL Pescara P.O., Pescara, Italy; 5 Italian Foundation of Medical Oncology (Fondazione AIOM), Milan, Italy; 6 Casa Di Cura Piacenza, Internal Medicine and Oncology, Via Morigi 41, 29121 Piacenza, Italy; 7 Department of Medical Oncology, IRCCS Ospedale Sacro Cuore Don Calabria, Negrar Di Valpolicella, Verona, Italy; 8 Italian Federation of Volunteer-Based Cancer Organizations (FAVO), Rome, Italy; 9 Italian Association of Cancer Patients, Relatives and Friends (Aimac), Rome, Italy; 10 Associazione Salute Donna Onlus, Milan, Italy; 11 Primary Care Physician, Bioethicist and Professor of Narrative Medicine, Ancona, Italy; 12 Scientific Journalist for Umberto Veronesi Foundation and Corriere Della Sera, Milan, Italy; 13 WHIN (Web Health Information Network), Milan, Italy; 14 Risk Management and Health Technology Innovation Unit, Department of Staff, AOU Delle Marche, 60126 Ancona, Italy; 15 Department of Surgical, Oncological and Oral Sciences, Section of Medical Oncology, University of Palermo, Palermo, Italy; 16 Italian National Institute of Health, Rome, Italy; 17 Medical Oncology Division and Breast Unit, Senatore Antonio Perrino Hospital, ASL Brindisi, Brindisi, Italy

**Keywords:** Cancer, Patient, Media, Scientific community, Journalism, Social network

## Abstract

**Aim:**

To identify barriers between health and communication in oncology in order to promote the best possible practice. The areas of communication to be focused on are communication directly with the patient, communication within the scientific community, and communication with the media.

**Material and methods:**

A working group including eminent experts from the national mass media, healthcare system, and patients’ advocacy has been established on behalf of the Italian Association of Medical Oncology (AIOM), with the aim of developing suitable recommendations for the best communication in oncology.

A literature search has been conducted selecting primary studies related to the best practices applied to communication in oncology. Subsequent to having identified the most representative statements, through a consensus conference using the RAND/University of California Los Angeles modified Delphi method, the panel evaluated the relevance of each statement to support useful strategies to develop effective communication between oncologist physicians and patients, communication within the scientific community, and communication with media outlets, including social media.

**Results:**

A total of 292 statements have been extracted from 100 articles. Following an evaluation of relevance, it was found that among the 142 statements achieving the highest scores, 30 of these have been considered of particular interest by the panel.

**Conclusions:**

This consensus and the arising document represent an attempt to strengthen the strategic alliance between key figures in communication, identifying high-impact recommendations for the management of communication in oncology with respect to patients, the wider scientific community, and the media.

**Supplementary Information:**

The online version contains supplementary material available at 10.1007/s00520-024-08786-8.

## Introduction

During the past few decades, the relationship between healthcare professionals and patients has changed dramatically. The latter has gradually taken on an increasingly important role in the process of the therapeutic alliance with the physician, characterized by a common purpose of intent and objectives within the treatment path. Establishing a relationship of defined responsibilities and tasks between the two parties is an essential step for the success of treatments, and this is particularly true in oncology [1]. From this point of view, dialogue plays a decisive role, not only for the oncologist but for all the people called upon to deal with patients (nurses, case managers, clinicians, and surgeons).

In recent years, the need to “promote public health communication” has been taken into consideration in the Italian National Health Plan by involving healthcare providers, the media, and the general public. It is also worth mentioning that the relationship between the three aforementioned groups is still rapidly changing as a result of the recent COVID-19 pandemic [2].

Communication in clinical practice might present difficulties and challenges, and this is particularly true in oncology. In this context, it appears essential that there is a communication synergy between healthcare professionals and patients which will serve to counter disinformation and fake news. However, physicians often have to share difficult decisions and break bad news to patients and families [1]. Furthermore, nowadays, the Internet gives the general public the possibility to access an extraordinary amount of information. Patients and caregivers can find additional information about cancer and clarifications on the diagnosis and prescribed treatments through the Internet [3, 4]. Yet it is known that not all the information available on the web is accurate and precise, and in some cases, extremely misleading data can be found. In oncology, misinformation is perhaps more harmful than in other fields because it impacts public health and potentially impacts fragile patients who could ultimately make incorrect decisions about their treatment path [5]. The quality of clinician-patient cancer communication is vital to cancer care and survivorship [6]. Physicians should be able to establish good communication and a relationship of trust with the patient as this would improve the patient’s well-being [6].

Even on websites supported by organizations officially recognized as reliable, a misinterpretation by an inexperienced reader could generate anxiety and confusion [7, 8]. Therefore, to support patients, healthcare professionals should learn to help them efficiently, providing them with the right information and addressing the concerns of both patients and caregivers [1]. For this reason, good communication skills should lead to the following:

- Ensuring therapeutic adherence through a trusting and efficient relationship
- Ensuring support at every stage of the disease
- Providing suggestions to improve the quality of life
- Having a positive impact on the patient’s mood

Furthermore, a comprehensive communication of methods and results of the scientific research is needed, since an inadequate and inappropriate mediation of this information may result in misleading and misinterpretations [9].

Nowadays, the search for answers via the Internet and within blogs, social networks, content-sharing platforms, and discussion forums gives access to a plethora of quickly accessible information about health, which is often not entirely accurate and sometimes even mendacious. In this regard, it is essential to help patients use the media available correctly to ensure that the information provided is properly filtered and certified, avoiding misleading information [10].

This manuscript represents the result of a substantial and demanding work promoted for the first time in this area by the Italian Association of Medical Oncology (AIOM). A qualified panel of healthcare experts, together with journalists’ and patients’ associations, have identified through the evaluation of available literature concerning the aforementioned topics, a set of good practices derived from the best scientific evidence and from the opinion of the experts themselves, who were consulted about the best strategies to apply in the relationship between communication and health.

## Material and methods

A working group coordinated by the AIOM society including eminent experts from the fields of National mass media, patients’ advocacy, and healthcare was formed in January 2023. The main aim of the consensus was to set up recommendations about “Communication in Oncology” under the following three major topics:

- Communication with the patient
- Communication within the scientific community
- Communication with the media and via the media

Eighteen experts coming from the AIOM board of directors and the main national newspapers (i.e. “Corriere della Sera”) and scientific societies (i.e., Italian Society of Psycho-Oncology, Italian Society of Narrative Medicine, AIOM) were selected. No specific criteria were adopted in the selection of the panelists. One invited expert declined the proposal; meanwhile, seventeen participants, including opinion leaders and directors with a recognized academic and institutional background and expertise in the communication and healthcare field, accepted to be involved in the project. The multidisciplinary expert panel met via teleconference and corresponded through email. Based on the consideration of the evidence, clinical experience, and a formal consensus process, the authors were asked to contribute to the development of a series of recommendations, providing critical review and finalizing the document. Members of the Expert Panel were responsible for reviewing and approving the penultimate version of the document, which was then circulated for external review and submitted to an impacted journal.

**Fig. 1:**
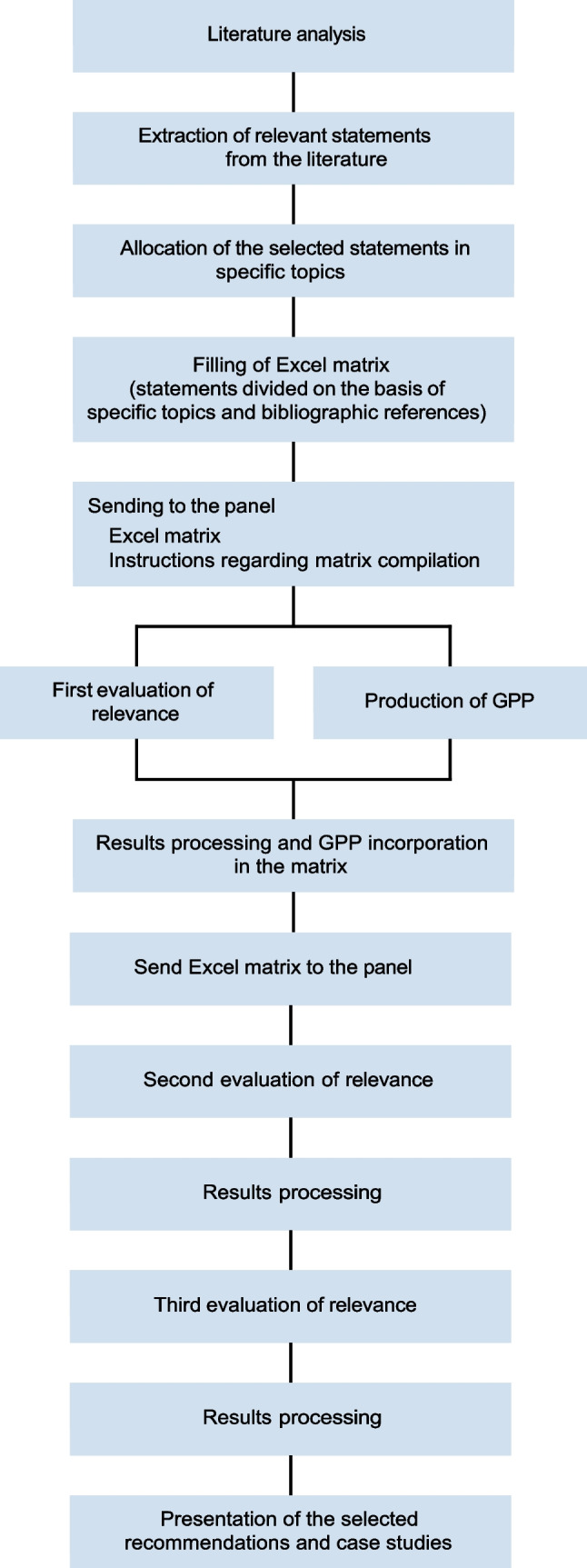
The modified version of Delphi methodology by RAND/UCLA developed by the working group. GPPs, good practice points; UCLA, University of California Los Angeles

A modified version of the Delphi methodology by RAND/University of California Los Angeles (UCLA) was employed as a consensus tool by participants [11] (Fig. 1). The original Delphi tool is a quick and structured method for obtaining opinions on a specific topic by a group of experts constituting the evaluation panel. The members of the group then evaluate a matrix containing statements, partly from the scientific literature, partly produced by the experts themselves through several rounds (i.e., good practice points—GPPs); each round is defined on feedback from the previous evaluation.

### Literature search

A literature search on PubMed has been conducted with the aim of selecting primary studies related to the best practices applied to communication within the scientific community, with the patients, and with the media. The search strategy was carried out in December 2022, using the following main search string: “communication” and “cancer” MESH terms. Eligibility criteria were as follows:

1. Studies investigating topics related to communication in oncology within the contexts of communication with patients, with the media, and among healthcare providers
2. Studies published in the English or the Italian language
3. Studies on healthy adult people (no pediatric issues)
4. Studies involving patients with solid cancer (no hematologic malignancies)
5. Studies published from January 2000 to December 2022
6. Studies involving Western countries (not low-income countries)
7. Other (study protocols, case studies)

### Statements selection

Two medical oncologists, independently, proceeded to do a preliminary detailed reading of the papers. They both extracted the best evidence and produced a series of statements, following a critical and objective assessment of the results of the documents found. Following a comparison of the selected items, with the support of a data manager, a list of statements was structured in an Excel format matrix linked to a set of information, such as bibliographic references (authors of the paper, title, journal and year of publication, and country where the study was conducted). Furthermore, the following major subtopics were identified for each topic:

1. Communication within the scientific community
  Research and communication
2. Communication with the patients
  General considerations
  Prognosis and diagnosis
  Experimental and standard treatment options
  End-of-life management
  Information
  Gender and sex-specific topics
  Caregiver
  Screening
3. Communication with the media
  Journalistic communication
  Web, social network, and data-sharing websites

**Fig. 2:**
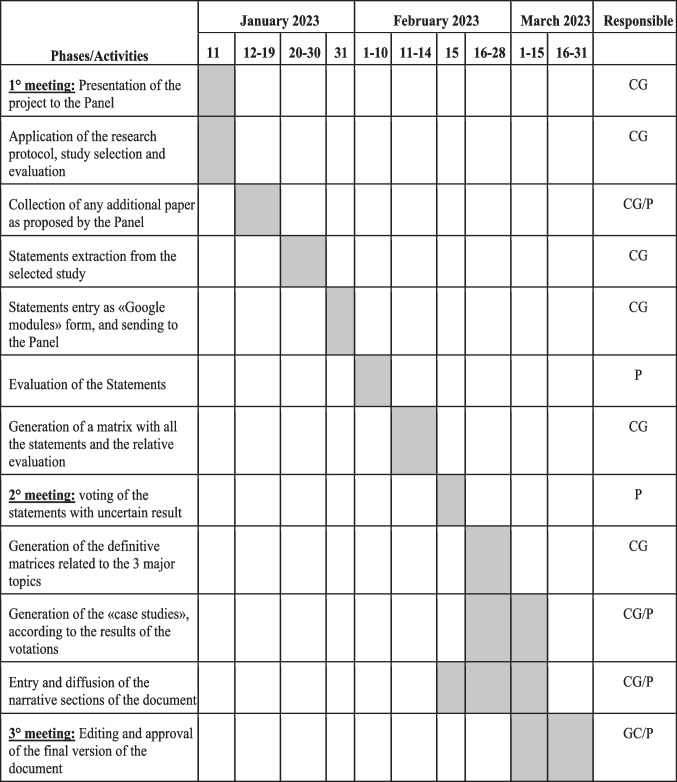
Gantt chart of the project. P: panel; CG: methodologic-scientific coordinating group

The Excel matrix was subsequently sent to the panel. The Gannt chart with the timeframe of this section of the project is detailed in Fig. 2.

### Relevance evaluation of the statements selected from the literature, additional recommendations, and case studies

The members of the panel reviewed the literature search results and evaluated the relevance of each statement to support useful strategies for overcoming communication barriers within the major areas identified. A modified version of Delphi methodology has been used for the evaluation: specifically, the panel members evaluated the relevance of good practices selected as follows:

1. First evaluation of relevance: individual independent assessment by each panel member of each statement proposed within the specific major topics. The judgment was expressed on a scale from 1 to 9, where 1 = certainly irrelevant, 9 = certainly relevant, and 5 = uncertain.
2. Second and third evaluation of relevance (with the possibility of group comparison): re-evaluation of intermediate judgments (band 4–6) was performed. Participants produced a report showing the results of the first evaluation for each recommendation. The discussion then focused on the areas of disagreement that might have emerged, and a second round of evaluation of relevance was done.
3. Data analysis: the scenarios were judged in agreement in which, after excluding the two outlying judgments from the analysis, the remaining judgments fell into any of the score ranges (1–3, 4–6, and 7–9), corresponding to the three levels of evaluation.

In addition to the compilation of the matrix according to the above criteria, participants were asked to provide additional recommendations to be referred to as GPP, attributed to the three predefined major topics, and then submitted to the panel (Fig. 1 and Data Supplement).

The presentations and the filled-in matrices were sent to the working group 3 days before the event so that the proposed recommendations could be included in the matrix related to the second round. Finally, in support of each specific topic addressed, the group deemed it appropriate to present some successful case studies. On the basis of the average evaluation of the various recommendations, these were then included in the final document. The Gantt chart with the timeframe of this part of the project is detailed in Fig. 2.

## Results

**Fig. 3:**
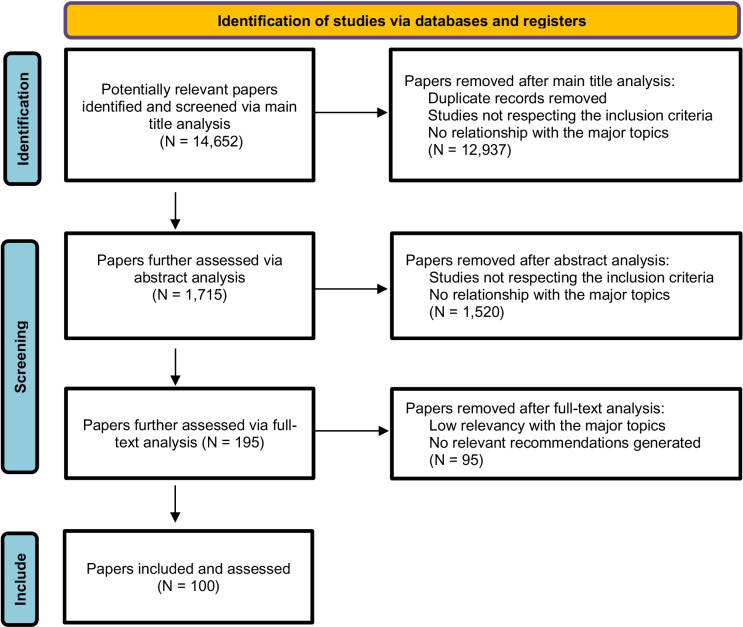
Algorithm of selected papers and recommendations

As displayed in Fig. 3, the authors initially identified 14,652 articles during the search; but through subsequent analytical steps, the articles that met the inclusion criteria and were judged eligible for producing statements were found to total 100. From these, 292 recommendations/statements were made, of which 44 were generated as GPP.

Following an evaluation of relevance by the panel of experts, it was found that 280 recommendations scored more than 7, 12 scored between 4 and 6.9, and no recommendation scored less than 4. Among the recommendations with the higher score, 142 scored between 8 and 9. Of these higher-scoring recommendations, 30 statements have been considered deserving of particular mention and relevance by the expert panel and have been listed in Table 1.

**Table 1:** Selected good practice points (GPPs) and literature recommendations among those with the highest score

Recommendations		Score
- Communication within the scientific community		
Research and communication		
1	News and information about new clinical trials should be disseminated, and the sources should be duly cited	9
2	All medical and pharmaceutical information should be free of manifest publicity and of benefit to interested parties	9
3	Any form of sensationalism, including innovative therapies, should be avoided, both in the terminology used and in any graphic support tools. [1, 12]	9
4	It is necessary to make the contents and results of scientific research understandable and interesting while maintaining their truthfulness and using understandable language	9
5	Communication professionals should produce clear and concise messages in an attempt to facilitate public understanding. [13]	8
6	Moreover, they should also produce clear and concise messages in an attempt to facilitate public understanding. [14]	8
- Communication with the patients		
General considerations		
7	Most cancer patients want a better dialogue with clinicians	8
Diagnosis and prognosis		
8	It is advisable to avoid arousing excessive fear or excessive hope in communication	9
9	Effective communication increases people’s satisfaction, reduces distress, promotes faster recovery and improves pain control, adherence to treatment and quality of life. [33, 34]	8
10	It is desirable to use simple language to describe cancer and its treatment. Too much technical language can have a negative impact on informed consent, adherence to therapy and screening. [8, 35]	8
Standard and experimental treatment options		
11	Providing information about treatment options gradually and constantly verifying the understanding of the discussed, can help improve the assimilation of the notions by the patient and the therapeutic alliance with the doctor. [1]	9
12	Appropriately clarifying the purposes of treatment, the expected outcome and the inconveniences potentially related to each treatment option can facilitate the understanding and assimilation of information by the patient and improve the therapeutic alliance with the doctor. [1]	8
13	More patient information during the informed consent process can help manage your expectations regarding the measurement and probability of the expected benefits of genomic sequencing analysis. [16]	8
End of life		
14	Early communication on palliative care and end-of-life issues is recommended in the case of diagnosis of incurable malignant diseases with limited life expectancy. [36]	8
15	Preventing and treating end-of-life pain and discomfort between patients and their loved ones is appropriate and an empathic response in this regard is important, as well as being familiar with patients and informing them about the local resources available to provide support to them and their loved ones, also in order to avoid the continuation of aggressive, burdensome and expensive treatments. [1, 37]	8
Information		
16	In order to reduce the risk of a perception not adhering to reality about the curability of the disease of a patient suffering from metastatic pathology, the doctor should take into account sex, education, clinical conditions, work and the country of origin of the same in order to improve medical-patient communication and patient care. [38]	8
Gender identity and sex-related topics		
17	Avoiding preconceptions about sexual orientation and gender identity can help make all patients feel accepted and empowered. Likewise, doctors should use non-critical language when talking about sexuality and sexual orientation. [8]	8.5
Caregiver		
18	Family involvement can improve target-related communication in patients with advanced cancer. In this regard, the doctor should take into account the opinions of patients about the involvement of their family in the course of the disease, how the disease can affect the well-being of the family itself, as well as the family’s expectations about the purposes of processing [1, 39, 40]	8
19	Caregivers of cancer patients tend to make extensive use of social networks in order to find useful information for the management of loved ones. It is essential that medical and paramedical personnel understand this and appropriately direct the caregiver towards adequate sources of information, trying to reduce the risk of misinformation. [41]	8
Screening		
20	It is desirable to implement personalised communication strategies (e.g. dedicated telephone line) of appointments and the purposes of cancer screening (not only breast) with particular regard to those groups of subjects potentially at high risk, where communication by correspondence alone may be insufficient. [42]	8
- Communication with the media		
Journalistic communication		
21	The journalist should ensure that the medical-pharmaceutical information is as correct as possible so as not to create false expectations	9
22	It is necessary to provide journalists with effective, simple and concise information on therapies and reference therapeutic innovations	8
23	Physicians and journalists must work together with respect for patients' dignity and the right/duty to inform	9
24	It is necessary to adapt the language and the channel used to the characteristics of the audience to be reached. [25]	8
Web, social networks, data-sharing websites, and platforms		
25	It is necessary to safeguard patients from social networks refuting fake news	8
26	Social networks can provide cancer patients with emotional support and information to improve their quality of life. [6]	8
27	Among the content sharing platforms, YouTube seems to be the most popular and widespread source of health information for patients with different clinical conditions. It is therefore desirable that healthcare professionals approach this type of media in order to ensure reliable and comprehensive information to the patient. [14]	8
28	Health authorities must make available a multiplicity of relevant information through multiple channels. [25]	8
29	Health authorities must also take an active role in combating misinformation on the web and on social media. [25]	8
30	It is necessary to acquire communication skills to inform and interact with patients through digital technology: how to listen/detect patients' needs, provide guidance in simple (understandable) and comprehensive language (don’t take anything for granted) to meet their information needs	8

## Discussion

Communication in cancer care is challenging. These recommendations, the first to be produced by a national scientific society like AIOM, summarize expert consensus on best practices in communication for clinicians caring for patients with cancer. Furthermore, different aspects of communication have been considered and defined in GPP.

Concerning communication within the scientific community, the panelists felt that news and information on clinical trials should be disseminated in a clear and objective way, avoiding sensationalism and producing concise messages in an attempt to facilitate public understanding. Given the traditional and Internet-based sources for medical research and healthcare-related news now available, now than ever, it is imperative that scientists know how to communicate their latest findings through the appropriate channels. The credible media channels are managed by working journalists, so learning how to package vast, technical research in a form that is appetizing and “bite-sized” in order to get their attention, is an art that needs to be cultivated [12–14].

Concerning communication with patients, the panel stressed the significant need for the patients to have a more balanced dialogue with clinicians and healthcare professionals about diagnosis and prognosis, to be conducted in understandable, jargon-free language [8]. In fact, many patients want to actively participate in their own healthcare decisions, and accessing reliable, accurate information is key to improving health literacy and allowing patients to be engaged in their care pathway. It is important for patients to have a full understanding of the implications of their disease at the time of diagnosis and during treatment. Patients do not always know what to ask and when to ask it or they potentially do not understand all of the information given by doctors and might not be fully aware of the subsequent implications in terms of diagnosis and prognosis [15].

The panel highlighted the important request for appropriate information about standard and experimental treatment options. For instance, it is known that cancer patients participating in precision oncology intervention research have largely unfulfilled expectations of the direct benefits related to their study participation [1, 16].

In addition, patients might not fully consider the long-term potential consequences of initiating a therapy or they may not be fully aware of the possible side effects and complications linked to treatment. On the other hand, a patient-clinician relationship could significantly affect patients’ reporting behaviors, which can potentially interact with other factors, including the severity of adverse events. It is important to engage oncology patients in medication safety self-reporting from home by enhancing health communication, understanding patients’ perceptions of severe events, and promoting patient activation. By addressing these efforts, healthcare providers should adopt a more patient-centered approach to enhance the overall quality and safety of oncological care [17].

Clinicians should develop skills to create a relationship with their patients based on trust. When clinicians understand who their patients are, their cancer treatment, and how they make decisions, patients feel more comforted. Consequently, clinicians understand that there are many gaps in health communication, and therefore, it is vital to create communication training programs to guarantee physicians are equipped to satisfy patients’ healthcare needs and improve their level of care. Furthermore, correct information generates knowledge, and when this knowledge becomes widespread, especially within the community, it contributes profoundly to prevention, which must be the main objective in the field of health [1]. Since communication skills training is a recognized important part of education in all oncology disciplines, specific training in breaking bad news should be required. To reach this aim, communication skills training should be based on sound educational principles, including and emphasizing skills practice and experiential learning using role-play scenarios, direct observation of patient encounters, and other validated techniques. It would be desirable for communication skills training to promote practitioner self-awareness and situational awareness related to emotions, attitudes, and underlying beliefs that may affect communication as well as awareness of implicit biases potentially affecting decision-making [18, 19].

Furthermore, early communication on palliative care and end-of-life issues is recommended and has been assessed as a GPP by the working group panel. Under this point of view, wide-ranging and innovative communication strategies and skills are required by clinicians to facilitate referral to early palliative care for cancer patients and their families. These themes might include using carefully selected and rehearsed language, framing in terms of symptom control, framing as an additive to patient care, selling the service benefits of early palliative care, framing acceptance of referral as an altruistic act, and adopting a phased approach to delivering information about palliative care [20]. It would be desirable that, besides clinicians, even clinical oncology nurses would acquire experienced communication skills focused on palliative care and end-of-life, including mentoring by expert interprofessional practitioners. This would strengthen the relationship between the healthcare team, patients, and caregivers [21].

With regard to the relationship between patients and healthcare professionals, an emerging notable request is to avoid preconceptions about sexual orientation and gender identity (SOGI). The transgender and gender-diverse population represents an underserved group across the cancer care continuum. On this issue, AIOM has recently published the results of two national surveys [22], showing that educational interventions and implementation of person-centric cancer policies are urgently needed. Personalized medicine is gradually emerging as a paradigm in oncology and incorporating sex-specific adjustments is unavoidable. This approach would ensure that scientific research considers gender differences and translates this knowledge into clinical practice within healthcare organizations. Ultimately, communication interventions dedicated to oncological diseases should also account for biological disparities among patients, while paying attention to cultural and social factors, as recently underlined by a consensus of the AIOM [23].

Finally, recommendations on media communication were made. Alongside the need for cooperation between healthcare professionals, journalists, and science communicators in order to disseminate effective information on cancer and improve the right/duty to inform, other noteworthy points concern the role of the Internet. While there are excellent freely available resources online, unfortunately, the Internet also contains information that can be ill-informed or even potentially dangerous. However, patients with cancer and their families are often challenged by fear and are desperate to hear about innovative or even miraculous treatment options, making them particularly vulnerable to pseudoscience and fake cancer news. The COVID-19 pandemic has illustrated the consequences of misinformation- and disinformation. The panelists mentioned the need to protect and safeguard patients from exposure to misinformation on the Internet and social media since combatting misinformation is a key part of effective communication. As a trusted source for healthcare advice, hospitals and healthcare providers can play an important role in addressing health misinformation. Beyond promoting timely, accurate, and accessible information, countering misinformation requires understanding the nature and scope of circulating falsehoods to address them effectively. In this regard, healthcare professionals and cancer patient advocacy organizations can often recommend reliable, accurate, and safe sources online, which patients and their families can access for trustworthy additional information about their cancer [6]. Particularly, healthcare providers (clinicians and nurses) might properly correct health misinformation on and off social media through a conceptual model implicating a first phase of acts of authentication and a second phase of acts of correction, ultimately using a variety of dissemination strategies [24].

Moreover, the working group felt that health authorities must also take an active role in combating misinformation [25]. In this regard, similarly to what was previously performed during the COVID-19 pandemic, the establishment of dedicated Misinformation Response Units to monitor messages containing dangerous misinformation presented on multiple media platforms, including social media, non-English media, international sites, and proliferating community forums, would be desirable [26]. Besides this example, multiple evidence-based tools and strategies might be adopted by healthcare providers and health authorities to effectively promote health information and challenge misinformation: from partnering with community groups and local organizations to build trust and mutual understanding, to develop high-quality, accessible health information in different digital formats, “debunking” misinformation already spreading in the information ecosystem or “prebunking” misunderstandings or misinformation before they become widespread so building resilience against belief in misinformation, adopting a form of communication based on storytelling and narratives in order to make information more interesting, understandable, believable, and persuasive [27–29]. Ultimately, it is important to understand and identify key mechanisms and actors in an “infodemic” (intended as the overabundance of good and bad information widely spreading via digital and physical information systems, that makes it difficult for people to make decisions for their health); infodemic is a reality even in the oncology setting and cannot be suppressed but need to be managed at every level [30]. In this respect WHO has put forward a competency framework and several training sessions for infodemic managers [31].

From an oncology perspective, it is now clear that a range of tools and strategies are needed in the battle against cancer, not only the fundamental and irreplaceable ones of medicine and scientific research.

Poor communication in cancer care could negatively affect the patient’s engagement and commitment as well as potentially have additional costs concerning psychological distress, unnecessary treatment, and indirect costs to the care system [32]. Good open communication must be a feature of every aspect of cancer care and is a noteworthy response to the issues surrounding communication emerging from these recommendations. This approach, which has been spreading in recent years and is the central theme of this paper, creates a strong doctor-patient bond and an important and progressive involvement of the patient and caregiver in the therapeutic process, with a series of advantages that are not just subjective, but also quantifiable.

This work presents some limitations. The selection of papers from the literature and the identification of the statements from the articles have been inevitably affected by the subjectivity of the authors who dealt with the selection; the heterogeneity of the papers found in the literature is another limitation. Moreover, the bibliographic research considered exclusively scientific publications in the medical field, excluding those coming from marketing and communication settings. However, this choice was made on the basis of the technical nature of the addressed issues: the idea was to start from indications coming from experts of the specific context of the application. Considering the multiplicity of stakeholders involved and the need for an integrated and multidisciplinary approach to the investigated topic, the chosen method has been the Consensus Conference, in the RAND/UCLA variant, which is widely used in the medical field. This methodology has never been used in studies related to social sciences; then, our work might be considered the first application in this field leading to interesting results. The RAND/UCLA method was helpful in managing the limitation related to the poor availability of data from randomized clinical trials and the general paucity of high-grade literature evidence in relation to the main aspects of communication herein discussed. Moreover, as recommended by the Rand/UCLA user’s manual, the relatively high number of multiple different professionals who were included in the Expert Panel might have limited the above-mentioned subjectivity in the selection of the studies [11].

## Conclusion

Communication in cancer care is challenging, and good open communication can improve every aspect of the cancer care pathway. The recommendations presented in this paper, which are the result of a widely comprehensive working group of AIOM, should be taken into consideration by oncology healthcare professionals to ensure their widespread application and from health authorities to encourage and support specific, organized approaches and training aimed at improving communication with patients and their families, which is crucially important for all concerned.

## Supplementary Information

Below is the link to the electronic supplementary material.

Supplementary file1 (XLSX 103 KB)

## Data Availability

No datasets were generated or analysed during the current study.
